# Successful application of virtual screening and molecular dynamics simulations against antimalarial molecular targets

**DOI:** 10.1590/0074-02760160207

**Published:** 2016-11-10

**Authors:** Renata Rachide Nunes, Marina dos Santos Costa, Bianca dos Reis Santos, Amanda Luisa da Fonseca, Lorena Sales Ferreira, Rafael Cesar Russo Chagas, Alisson Marques da Silva, Fernando de Pilla Varotti, Alex Gutterres Taranto

**Affiliations:** 1 Universidade Federal de São João Del-Rei, Laboratório de Química Farmacêutica Medicinal, Divinópolis, MG, Brasil; 2 Centro Federal de Educação Tecnológica de Minas Gerais, Departamento de Informática, Gestão e Design, Divinópolis, MG, Brasil; 3 Universidade Federal de São João Del-Rei, Núcleo de Pesquisa em Química Biológica, Divinópolis, MG, Brasil; 4 Universidade Federal de São João Del-Rei, Laboratório de Compostos Bioativos e Catalíticos, Divinópolis, MG, Brasil

**Keywords:** Plasmodium falciparum, plasmepsin-II, plasmepsin-IV, falcipain-II, PfATP6, virtual screening

## Abstract

The main challenge in the control of malaria has been the emergence of drug-resistant parasites. The presence of drug-resistant *Plasmodium sp.* has raised the need for new antimalarial drugs. Molecular modelling techniques have been used as tools to develop new drugs. In this study, we employed virtual screening of a pyrazol derivative (Tx001) against four malaria targets: plasmepsin-IV, plasmepsin-II, falcipain-II, and PfATP6. The receiver operating characteristic curves and area under the curve (AUC) were established for each molecular target. The AUC values obtained for plasmepsin-IV, plasmepsin-II, and falcipain-II were 0.64, 0.92, and 0.94, respectively. All docking simulations were carried out using AutoDock Vina software. The ligand Tx001 exhibited a better interaction with PfATP6 than with the reference compound (-12.2 versus -6.8 Kcal/mol). The Tx001-PfATP6 complex was submitted to molecular dynamics simulations in vacuum implemented on an NAMD program. The ligand Tx001 docked at the same binding site as thapsigargin, which is a natural inhibitor of PfATP6. Compound TX001 was evaluated in vitro with a *P. falciparum* strain (W2) and a human cell line (WI-26VA4). Tx001 was discovered to be active against *P. falciparum* (IC_50_ = 8.2 µM) and inactive against WI-26VA4 (IC_50_ > 200 µM). Further ligand optimisation cycles generated new prospects for docking and biological assays.

Malaria continues to the leading cause of mortality among all infectious diseases in the world; however, the current chemotherapeutic arsenal has limited clinical response owing to parasite resistance. The presence of different strains of drug-resistant *Plasmodium sp.* highlights the need for new antimalarial drugs.

Target identification is the first step in developing new antimalarial drugs. This has motivated the construction of a large databank of ligands and molecular targets, including data from the ZINC and Protein Data Bank (PDB). With this information, virtual screening (VS) techniques can be applied to search for the most promising ligands in the databank against one or more molecular targets (Westermaier et al. 2015).

VS is a large-scale docking methodology (Jaghoori et al. 2016). The aim of docking is to accurately predict the structure of a ligand within the constraints of a receptor binding site and to correctly estimate the binding strength. This methodology requires the preparation of ligands and molecular targets (Elokely & Doerksen 2013). Among several VS methodologies (Westermaier et al. 2015), AutoDock Vina and SwissDock are notable for accessibility, free cost, and user-friendly interfaces. These features have increased their use by several research groups, particularly in the case of AutoDock Vina (Jaghoori et al. 2016). AutoDock Vina performs stochastic optimisation to establish a binding conformation. Next, the Metropolis criterion is used to accept this or not. In addition, AutoDock Vina uses both knowledge-based potentials and empirical scoring functions. SwissDock is a web server dedicated to the docking of small molecules on target proteins. It is based on the EADock DSS engine and also has scripts set up for curating common problems and preparing both the target protein and the ligand input files (Grosdidier et al. 2011). The SwissDock website is http://www.swissdock.ch. In the context of these existing tools, our group has developed a VS software called *Octopus*. In contrast to other VS platforms, *Octopus* can perform VS on a pool of ligands against a set of molecular targets. *Octopus* prepares the ligands using the semi-empirical parametric method (PM) 7 implemented on MOPAC2012 (Stewart 2013). Next, the Gasteiger charges are added automatically for each ligand. Finally, *Octopus* carries out docking simulations of the prepared ligands against each molecular target set *via* AutoDock Vina.

The use of VS to explore the chemical space *via* databanks has a high computational cost. Thus, it is preferable to filter the ligand and molecular targets to address issues particular to Brazil, including neglected diseases such as malaria and dengue fever. In this context, our group has built two databanks in house - ligand and molecular target databanks called Our Own Chemical Collection (OOCC) and Our Own Molecular Targets (OOMT), respectively. The OOCC is used to search through the compounds sent by collaborators, and OOMT is evaluated by re-docking and druggability (Carregal et al. 2013). In other words, we have performed VS for compounds that can be obtained by close collaborators against molecular targets with available biological assays. This is similar to the approach that pharmaceutical companies use to identify the most promising active compounds (Leite et al. 2012).

The main focus of this study was to perform VS on selected targets present in OOMT. The reported technique could be applied to other molecular target databases. However, in evaluating a large number of biological targets, the main challenge has been to capture and integrate relevant information in an easily accessible format to identify and prioritise potential targets. Hence, to facilitate the integration and mining of emerging data and the identification and prioritisation of targets, an open access database was used to organise the targets by their related tropical disease pathogens. Tropical Disease Research [TDR targets (http://tdrtargets.org)] was used to shortlist candidate targets that are suitable for further investigation (Agüero et al. 2008). The TDR database has listed the targets of the main pathogens of tropical diseases, such as the mycobacteria *Mycobacterium leprae* and *M. tuberculosis*; protozoa *Leishmania major*, *Trypanosoma brucei*, and *T. cruzi*; apicomplexa protozoa *P. falciparum*, *P. vivax*, and *Toxoplasma gondii*; and helminths *Brugia malayi* and *Schistosoma mansoni*. This tool aims to bring together genome sequencing project data, structural protein data, and information on the target and its druggability.

Among the compounds in the OOCC, the pyrazol derivative [amino({5-phenyl-3-[(E)-2-phenylethenyl]-4,5-dihydro-1H-pyrazol-1-yl})methylidene]azanium (Tx001; Fig. 1) was found to have particularly promising pharmacological activity, exhibiting anticancer, antioxidant, antibacterial, antifungal, antidepressant, anti-inflammatory, antitumour, and analgesic properties (Sharma et al. 2014). There is a strong correlation between anticancer and antimalarial properties of drugs (Manohar et al. 2013). Thus, the pyrazol derivative’s anticancer activity motivated us to perform VS against *P. falciparum* molecular targets present in the OOMT. Four antimalarial molecular targets were included in the OOMT, including plasmepsin-IV (PDB: 2ANL), plasmepsin-II (PDB: 1LF3), falcipain-II (PDB: 3BPF), and the PfATP6 model (Guimarães et al. 2015).

**Fig. 1:**
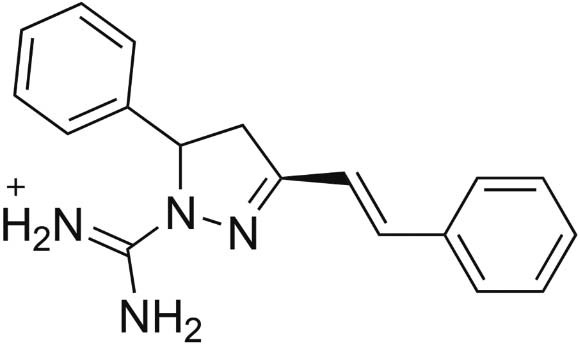
: the pyrazol derivative [amino({5-phenyl-3-[(E)-2-phenylethenyl]-4,5-dihydro-1H-pyrazol-1-yl})methylidene]azanium, which is herein referred to as Tx001 and subjected to virtual screening.

The maintenance of the erythrocytic cycle in malaria parasites relies on haemoglobin (Hb) degradation. Hb degradation occurs in an acidic compartment inside the parasite, namely, the digestive vacuole (DV). Hb degradation provides a source of amino acids for the parasite and maintains erythrocyte intracellular osmolarity during the erythrocytic cycle (Mauritz et al. 2009). Proteolytic enzymes are involved in Hb degradation, including aspartic proteases (plasmepsins) and cysteine proteases (falcipains; Moura et al. 2009). As Hb degradation is a vital process for the malaria parasite, plasmepsins and falcipains are considered potential antimalarial targets (Conroy et al. 2014).

In addition, PfATP6 is a sarco/endoplasmic reticulum Ca(^2+^)-ATPase (SERCA) present in the endoplasmic reticulum of *P. falciparum* (Krishna et al. 2014). Calcium signalling is associated with the regulation of a broad range of vital processes during the parasitic life cycle, including the synchronisation of the intraerythrocytic cycle (Hotta et al. 2000). In other words, these molecular targets are druggable and are useful in the drug discovery process.

In this study, we describe the successful case of Tx001, which is a potential compound identified *via* VS against four malaria targets: plasmepsin-IV, plasmepsin-II, falcipain-II, and PfATP6. Following VS, Tx001 was submitted for biological assay, which revealed that it possesses antimalarial activity in vitro, suggesting that it is a lead compound.

## MATERIALS AND METHODS

*Computational methods: evaluation of docking methodologies* - Initially, AutoDock Vina was evaluated by re-docking, which removes the crystallographic ligand with subsequent docking. The root mean square deviation (RMSD) of the heavy atoms in the crystallographic ligand and re-docked ligand should be less than 2.0 Å (Westermaier et al. 2015).

In addition, the receiver operating characteristic (ROC) curve and the area under the curve (AUC) (McGann 2011) were calculated for each molecular target. The active compounds 3, 5, and 6 were selected from ChemBL for plasmepsin-IV (2ANL), plasmepsin-II (1LF3), and falcipain-II (3BPF), respectively, and none for PfATP6 (Gaulton et al. 2012). Next, false-positive compounds were obtained from a directory of useful decoys using the DUD-E database (Mysinger et al. 2012). DUD-E could generate 150, 250, and 300 decoys for plasmepsin-IV, plasmepsin-II, and falcipain-II, respectively. Decoy and active ligands were submitted to the docking process in their default protonation states.

All docking simulations were carried out using AutoDock Vina (Trott & Olson 2010). The grid box was generated by AutoDock Tools (Morris & Huey 2009). The coordinates x, y, and z were defined according to Table I with spaced points of 1 Å centred on the ligand and an x, y, and z slice of 20 Å - This is sufficient to cover the binding site. The exhaustiveness parameter was set to eight.

**TABLE I:** Grid box size and position for all molecular targets

Coordinates (Å)			
	X	Y	Z
2ANL	54.924	13.448	25.686
1LF3	16.215	6.850	27.605
3BPF	-36.87	31.066	-47.069
PfATP6	-5.142	-48.212	8.979

*Preparation of molecular targets and ligand* - Initially, the druggability of the molecular targets was checked using the TDR database (Crowther et al. 2010). Afterwards, the targets were selected based on their biological role in *P. falciparum* and the availability of their 3-D crystallographic structures. The 3-D structures were retrieved with PDB codes 2ANL, 1LF3, and 3BPF with resolutions of 3.30, 2.70, and 2.90 Å, respectively. PfATP6 was obtained using a previous comparative modelling method (Guimarães et al. 2015). Next, the target protonation state was adjusted to pH 4.0 for plasmepsin-II, plasmepsin-IV, and falcipain-II to simulate the food vacuole environment. The pH was adjusted to 7.4 for PfATP6, simulating the membrane environment using the module PROPKA from an academic version of Maestro software.

Alternatively, the 3-D structure of Tx001 was generated using the MarvinSketch program, with its protonation state adjusted to pH 4.0. It was then refined with the MOPAC2012 program using the semi-empirical PM7 (Stewart 2013). The ligand Tx001 was oriented towards the binding site according to the grid box (Table I).

*Docking studies* - Rigid and flexible docking were performed with the AutoDock Vina program (Trott & Olson 2010). After the rigid re-dock, the amino acid residues of the binding sites in contact with the crystallographic ligand were chosen for flexible docking (Table II).

**TABLE II:** Residues set for flexible docking

2ANL	1LF3	3BPF	PfATP6
Asp34	Ile14	Gln36	Ile251
Gly36	Met15	Cys42	Leu253
Ile75	Ile32	Trp43	Phe254
Tyr77	Asp34	Leu72	Gln257
Gly78	Gly36	Asn81	Leu258
Leu131	Tyr77	Gly82	Ile261
Asp214	Val78	Gly83	Ile748
Thr217	Ser79	Leu84	Ile752
Val292	Ile123	His174	Asn755
Ile300	Tyr192		Ile756
	Asp214		Val759
	Ser218		Phe763
			Leu815
			Ile816
			Leu821
			Tyr824
			Ile825

*Molecular dynamics (MD) simulation of the Tx001-PfATP6 complex* - Initially, the previously built model (Guimarães et al. 2015) was evaluated using the Ramachandran plot implemented on Procheck software and Assessing Protein Structures with a Non-local Atomic Interaction Energy (ANOLEA; Melo & Feytmans 1998). The Ramachandran plot and ANOLEA evaluate structural and packing quality, respectively.

The unsuccessful search for active ligands for PfATP6 on ChemBL suggested that MD simulations should be performed to identify the false positive (Westermaier et al. 2015). In other words, it was not possible to evaluate PfATP6 *via* an AUC value. The Tx001-PfATP6 complex was subjected to MD simulation in vacuum implemented on an NAMD program over 35 ns. The CHARMM36 all-atom and CGenFF force fields were used for PfATP6 and Tx001, respectively. A time step of 1 fs was used, with all forces being evaluated at every step. A cut-off of 16 Å for both electrostatic and van der Waals calculations was used. Smoothing functions were applied to these interactions with force switching from 14-18 Å. Langevin dynamics were used to hold the temperature constant, and the Langevin coupling coefficient was 1/ps. Finally, the equilibrium structure was submitted to 5000 steps of energy minimisation under a conjugated gradient.

*Analysis of results* - DS Visualizer v.4.1 (Accelrys Software Inc., USA) was used to show the docking results for different binding conformations - this establishes the best molecular target of the compound. Plots of potential energy variation and hydrogen bond formation frequency throughout the MD simulations were generated using the Visual Molecular Dynamics program (VMD). Moreover, the ligand’s conformity to Lipinski’s ‘rule of five’ and druglikeness were evaluated using the DataWarrior program.

*In vitro culture of P. falciparum and in vitro antimalarial tests* - Antimalarial activity was evaluated in vitro in *P. falciparum* culture, as described below. The CQ-resistant W2 strain was cultivated in human type A+ red blood cells diluted at 5% haematocrit in RPMI-1640 medium supplemented with 10% human plasma, 0.36 mM hypoxanthine, 0.10 M glucose, 20 mM HEPES, and 2 mM glutamine. *P. falciparum* culture was maintained at 37ºC in a desiccator. For antimalarial tests, cultures containing 10-20% of ring stage parasites were synchronised with 10% sorbitol and the haematocrit was adjusted to 1%. Then, 180 µL of culture containing 1% ring stage parasites was added to 96-well plates followed by the addition of 20 µL of the test compound in a previously defined concentration (100-0.10 µM). All the tests were performed in triplicate. After 48 h incubation at 37ºC parasite growth was determined using a blood microscopic exam. The parasitaemia was assessed by counting the total number of infected cells in a sample of 1,000 red blood cells at an immersion objective of 100×. Antimalarial activity was expressed as the mean of the half-maximal inhibitory dose (IC_50_) and compared with that of the drug-free controls. Curve-fitting was performed using Origin 8.0 software (Origin Lab Corporation, Northampton, MA, USA). All experiments were performed in triplicate.

*Cytotoxicity assay* - The cytotoxic effect of the Tx001 was assessed against WI-26VA4, a lung fibroblast human cell line (ATCC CCL-95.1). The cell viability was determined using an MTT colorimetric assay. Briefly, the method consisted of plating 1 × 10^6^ cells in 96-well microplates, in which they were homogenised in RPMI 1640 medium supplemented with foetal bovine serum (FBS) and penicillin-streptomycin antibiotics. Then, the microplates were incubated overnight at 37ºC and under 5% CO_2_, followed by the addition of the pyrazol derivative solubilised in DMSO 0.1% (v/v). Negative control groups constituted cells without treatment. Five serial dilutions (1 : 10) were made from stock solution (10 mg mL^-1^) using RPMI supplemented with 1% FBS. Cell viability was evaluated after incubation for 48 h by removing and discarding the medium and adding 100 μL of MTT 5%, followed by 3 h of incubation. After incubation, the supernatant was removed and discarded and the insoluble formazan product was dissolved in DMSO. The optical density (OD) of each well of the 96-well plates was measured using a microplate spectrophotometer at 550 nm. The OD of the formazan formed in untreated control cells was defined as 100% cell viability. All experiments were performed in triplicate. The results were expressed as the mean of the IC_50._

A selectivity index (SI), corresponding to the ratio between the cytotoxicity and antimalarial activity of the compound, was calculated as follows:

IS = IC_50_ WI-26VA4 / IC_50_ *P. falciparum* W2

## RESULTS AND DISCUSSION

*Evaluation of docking methodologies* - AutoDock Vina was adopted for the VS experiments because of its speed and accuracy. However, in general, docking methodologies are structure-dependent (Elokely & Doerksen 2013). Thus, we evaluated AutoDock Vina by RMSD, AUC calculations, and MD simulations. The molecular targets were evaluated in terms of their druggability through TDR as well. The druggability index ranges from 0 to 1. Plasmepsin-II, plasmepsin-IV, and falcipain-II were found to have druggability indices of 0.8, 0.8, and 0.6, respectively. In addition, the druggability of PfATP6 is under study by our group. These data suggested that these molecular targets were suitable as targets for drug development.

**Fig. 2:**
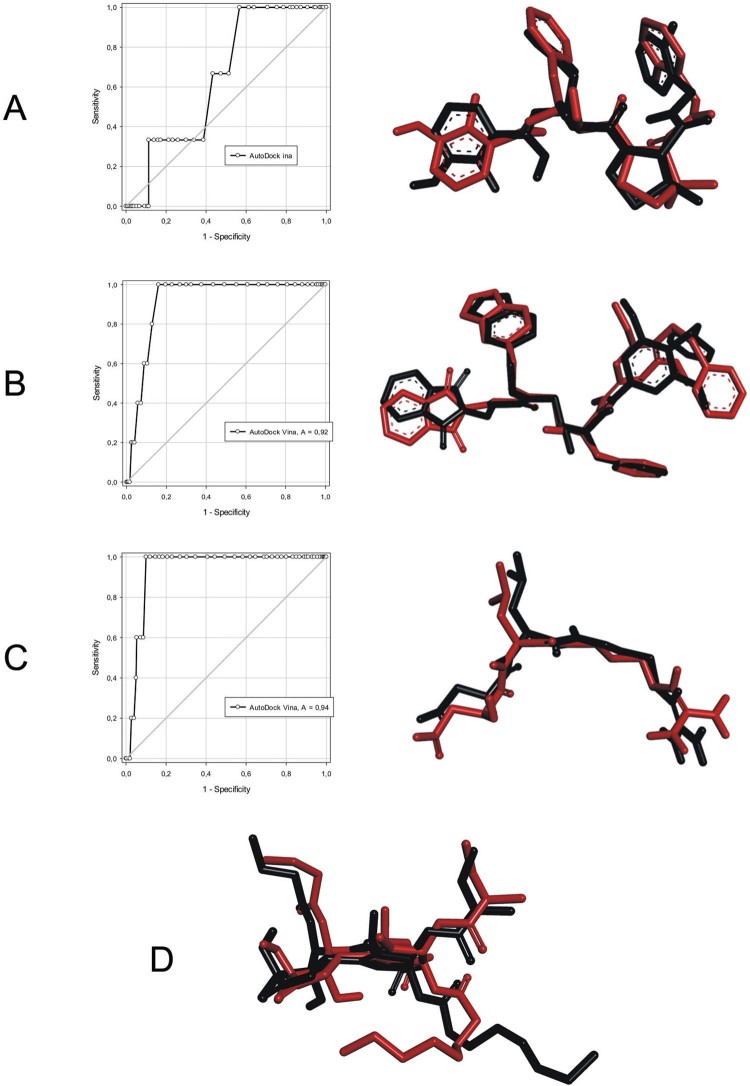
: re-docking using AutoDock Vina of (A) plasmepsin-IV (2ANL), (B) plasmepsin-II (1LF3), (C) falcipain-II (3BPF), and (D) PfATPase. Area under the receiver operating characteristic curve values of 0.64, 0.92, and 0.94 were obtained for plasmepsin-IV, plasmepsin-II, and falcipain-II, respectively. The hydrogen atoms are omitted for better visualisation. Colours red and black represent the re-docking and crystallographic structures, respectively.

Initially, the re-docking methodology was performed with AutoDock Vina. This re-docking showed the structural differences in the crystallographic ligand and docked ligand (Fig. 2). In general, AutoDock Vina could reproduce the crystallographic conformation for all molecular targets with RMSD values less than 2.0 Å (Westermaier et al. 2015). The best result was obtained for plasmepsin-IV (RMSD 0.25 Å). This ligand is peptide-like with a non-rotatable amide moiety, which improves the results. However, thapsigargin - a natural inhibitor of PfATP6 - has a flexible alkyl side chain, which minimises AutoDock Vina’s ability to reproduce the crystallographic structure (RMSD 1.88 Å). Similarly, plasmepsin-II (1LF3) has a peptide-like crystallographic ligand, which maintains the re-docking structure to be similar to the crystallographic structure. Finally, even though falcipain-II (3BPF) has a peptide-like ligand, it was found to have 10 rotatable bonds. This further decreases the performance of the methodology.

The ROC curve is a graphic plot that uses a binary classiﬁcation system to discriminate between active and inactive compounds. This graph shows sensitivity on the y-axis, with a maximum true positive rate of 1, and specificity on the x-axis, with the false-positive rate shown as 1 - specificity (Kellenberger et al. 2008). The quality of a probabilistic classification can be measured by the AUC, which measures how well the ROC curve separates two classes without a threshold decision value of reference. AUC values close to 1 mean that the compounds could be classified with an accuracy approaching 100%; whereas an AUC value of less than 0.5 indicates a random process (Fawcett 2006). In addition, the ROC methodology has a 95% confidence interval. We obtained AUC values of 0.64, 0.92, and 0.94 for plasmepsin-IV, plasmepsin-II, and falcipain-II, respectively. In other words, these three targets are within an acceptable range (Fig. 2); i.e. greater than 0.5 for plasmepsin-IV and close to 1 for plasmepsin-II and falcipain-II. Therefore, these results showed that AutoDock Vina can discriminate true positives from false positive.

*Docking studies* - AutoDock Vina was used to dock Tx001 against four enzymes. Table III shows the binding energies between the ligand Tx001 and plasmepsin-IV, plasmepsin-II, falcipain-II, and PfATP6 in both the rigid and flexible approaches. The positive ∆ values show that the ligand can bind more efficiently to molecular targets than to its own crystallographic ligand. VS has demonstrated that Tx001 can bind to PfATP6 in both the rigid and flexible approaches. In conclusion, this finding suggests that PfATP6 is a potential molecular target for Tx001. These data identify a lead compound *via* an optimisation process.

**TABLE III:** Binding energy (Kcal/mol) between the compound and crystallographic ligand against plasmepsin-IV, plasmepsin-II, falcipain-II, and PfATP6, using the software AutoDock Vina in flexible and rigid approaches. The ∆ was calculated *via* the difference in binding energy between the crystallographic and computational data for Tx001

	Flexible docking				Rigid docking			
	2ANL	1LF3	3BPF	PfATP6	2ANL	1LF3	3BPF	PfATP6
Tx001	-10.1	-10.4	-8.0	-12.2	-8.1	-8.6	-6.7	-8.6
Crystallographic	-12.7	-12.1	-8.1	-6.8	-12.5	-17.9	-6.8	-7.7
∆	-2.6	-1.7	-0.1	5.4	-4.4	-9.3	-0.1	0.9

**Fig. 3:**
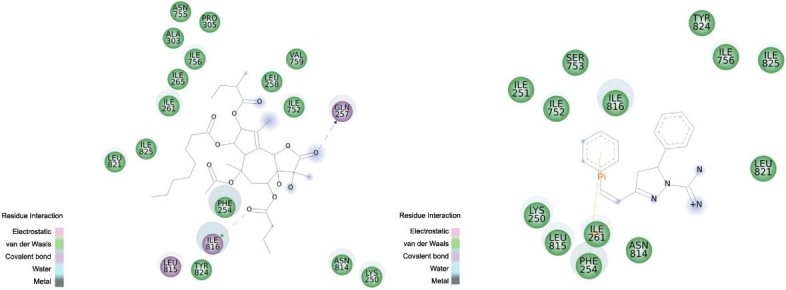
: PfATP6 binding site. (A) Thapsigargin; (B) ligand Tx001.

PfATP6 was selected as a molecular target for Tx001. This enzyme can recognise ligands principally by van der Waals interactions that characterise a hydrophobic pocket (Fig. 3). In addition to hydrophobic interactions, thapsigargin complexes with PfATP *via* electrostatic interactions through Gln257, Leu815, and Ile816. Tx001 displays additional π-stacking interactions with Phe254. Fig. 3 shows that thapsigargin interacts with Lys250, Phe254, Gln257, Leu258, Ile261, Ile265, Ala303, Pro305, Ile752, Asn755, Ile756, Val759, Asn814, Leu815, Ile816, Leu821, Tyr824, and Ile825, while Tx001 complexes with Lys250, Ile251, Phe254, Ile261, Ile752, Ser753, Ile756, Asn814, Leu815, Ile816, Leu821, Tyr824, and Ile825. Both shared some of the amino acids at the binding site: Lys250, Phe254, Ile261, Ile752, Asn814, Leu815, Ile816, Leu821, Tyr824, and Ile 825.

Finally, Tx001 was evaluated in terms of Lipinski’s rules and for its druglikeness using the DataWarrior program (Jorgensen 2004) (Table IV). An important strategy to define the potential antimalarial activity of a compound is based on its physicochemical properties (Leeson & Springthorpe 2007). Thus, Lipinski’s ‘rule of five’ and fragment-based druglikeness were estimated for Tx001. As seen in Table IV, there were no violations of Lipinski’s rule by the lead compound. In addition, the Tx001 had druglikeness of 2.76 calculated by DataWarrior. Fragment-based druglikeness scores the fragments of the ligand using a list of fragments from 15,000 commercial compounds. The majority of the commercial drugs have a druglikeness score close to two. This compound showed Lipinski’s rule features similar to those of antimalarial drugs such as chloroquine. Furthermore, DataWarrior did not report any mutagenic, tumorigenic, or irritant activity, indicating that the compound has a favourable druglikeness profile.

**TABLE IV:** Log P, molecular weight (MW), and the number of hydrogen bond acceptors (HBAs) and hydrogen bond donors (HBDs) were calculated using the DataWarrior program

	Log P	HBA	HBD	MW (g/mol)
Lipinski´s Rule	< 5	< 10	< 5	< 500
DataWarrior	3.33	4	2	291.38

**Fig. 4:**
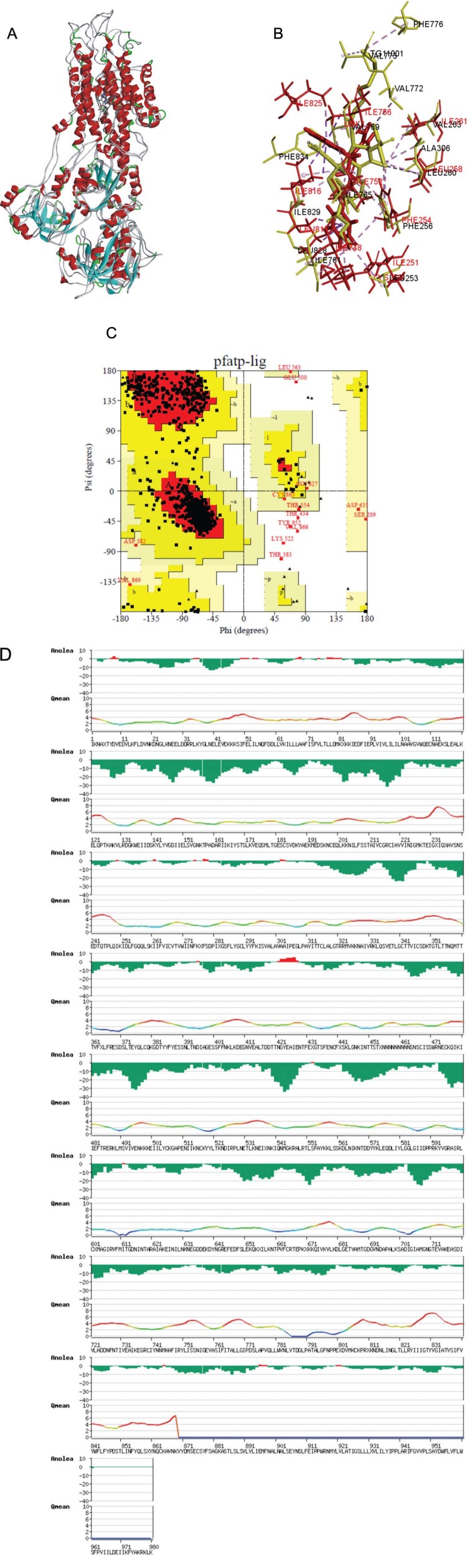
: evaluation of the PfATP6 model. (A) Superimposition of PfATP6 model and 1WIO template; (B) a close view of the binding sites of PfATP6 (red) and 1WIO (yellow); (C) Ramanchandran plot. Colours red, yellow, and white represent the most favourable, allowed, and disallowed regions, respectively; (D) Non-local Atomic Interaction Energy (ANOLEA). The y-axis shows the energy for each amino acid (aa) of the protein chain. Negative (green) or positive (red) energy values represent a favourable or unfavourable energy environment of a given aa, respectively.

*MD simulation of the Tx001-PfATP6 complex* - Initially, the previously built PfATP6 model (Guimarães et al. 2015) was evaluated by RMSD, a visual inspection of the binding site, Ramachandran plot, and ANOLEA. As a result, PfATP6 was built using 2O9J, 3BA6, and 1IWO as templates, with 43%, 49%, and 43.5% identity, respectively (Fig. 4A). The binding site of the model was therefore conserved, keeping amino acids similar to those of template 1WIO (Fig. 4B). The Val772, Val773, and Phe776 highlighted in the picture due the side chain of thapsigargin. The Ramachandran plot (Fig. 4A) showed that 87.8% and 10.6% in the most favourable and allowed region, respectively. According to the Ramachandran plot (Fig. 4C), using the template 1IWO with 3.1 Å resolution, a good model generally features 90% in the most favourable region. Moreover, the amino acids in the disallowed region are not part of the binding site. In addition, in general, ANOLEA (Fig. 4D) showed that the heavy atoms of the model had favourable energies, as calculated using the atomic empirical mean force potential (green). This report showed that the docking approach can be applied with success using a transmembrane receptor model, such as G-protein-coupled receptors. In addition, models which share 50% of identity with templates are sufficiently accurate to be used in a drug design context. In other words, our transmembrane model of PfATP6, which was built using three different templates and thoroughly evaluated, is suitable for use in docking simulations.

**Fig. 5:**
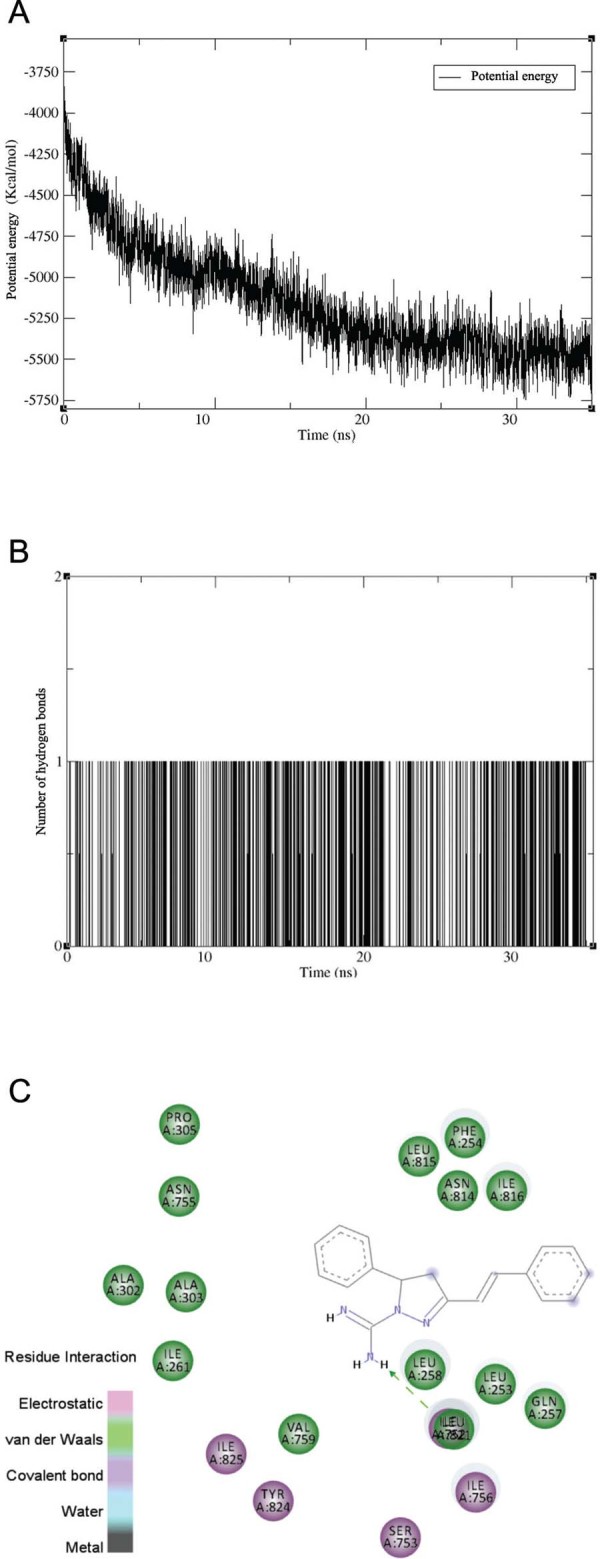
: molecular dynamics (MD) simulation of the Tx001-PfATP6 complex for 35 ns. (A) Potential energy; (B) number of hydrogen bonds that occurred during the simulation ranging from 1 to 3; (C) 2D diagram of intermolecular interactions between Tx001 and PfATP6.

The Tx001-PfATP6 complex was submitted to MD simulation in an implicit solvent model to examine the flexibility and conformational variations of ligand Tx001 complexed with PfATP6 over 35 ns. The potential energy of the system decreased during simulation to around -5500 Kcal/mol, achieving equilibrium in 30 ns (Fig. 5A). Tx001 was complexed with the molecular target during the entire process through electrostatic and van der Waals intermolecular interactions. It is worth noting that hydrogen bonds are the most important specific interactions in biological systems, and one hydrogen bond was maintained between the guanidinium moiety of Tx001 and Ile752 throughout the simulation. After 35 ns of MD simulation, Tx001 interacted with residues Leu253, Phe254, Gln257, Leu258, Ile261, Ala302, Ala303, Pro305, Ile752, Ser753, Asn755, Ile756, Val759, Asn814, Leu815, Ile816, Tyr824, and Ile825 (Fig. 5C). Finally, Tx001 and thapsigargin were found to share interactions with Phe254, Gln257, Leu258, Ile261, Ala303, Pro305, Ile752, Ile756, Val759, Asn814, Leu815, Ile816, Tyr824, and Ile825.

After VS, the Tx001 sample was used in a biological assay, and it showed antimalarial activity. In summary, VS helped in finding a lead compound. Our results showed similar binding sites for Tx001 and thapsigargin, indicating a similar mode of action as well. Thapsigargin inhibits PfATP6, promoting an efflux of calcium from the endoplasmic reticulum to the cytoplasm. *P. falciparum* trophozoites use intracellular calcium signalling for parasite development (Hotta et al. 2000). Thus, compounds that interfere with parasite calcium homeostasis could provide a new strategy in the design of new antimalarial drugs.

*Biological activity* - The antimalarial activity of compound Tx001 was evaluated in vitro against a *P. falciparum* chloroquine-resistant strain (W2). Results indicated that compound Tx001 was active against *P. falciparum*, with an IC_50_ = 8.2 µM (Table V). In parallel, no cytotoxic activity was exhibited against a WI-26VA4 fibroblast human cell line. Compound Tx001 presented a high selectivity index (> 24.4). Therefore, the development of fundamental properties of a new antimalarial candidate is a crucial factor ensuring the efficiency of the final drug. In this sense, a set of criteria have been suggested to guide the selection of a potential compound against malaria (Katsuno et al. 2015). Based on the in vitro activity, these criteria include: (i) an IC_50_ < 1 μM against resistant strains of *Plasmodium* and (ii) a SI greater than 10-fold. The preliminary results of compound Tx001 showed an SI within the postulated range. Further development of compound Tx001 now relies on increasing its antimalarial potency.

**TABLE V:** Antimalarial activity in vitro, cytotoxicity and selectivity index (SI) of Tx001

Compound	IC_50_ (µM) ± SD		
	*Plasmodium falciparum* W2	WI-26VA4	SI
Tx001	8.2 ± 0.04	> 200	> 24.4
Chloroquine	0.38 ± 0.015	> 200	> 526.3

SD: standard deviation.

Molecular modelling techniques such as comparative modelling, docking, molecular dynamics, and virtual screening have been used as tools in the development of new drugs. Such techniques identify drugs based on molecular targets. They simulate and predict toxicity, activity, bioavailability, and effectiveness. In this study, we evaluated the accuracy of a docking methodology with four molecular targets using RMSD and AUC. In all cases, AutoDock Vina exhibited sufficient accuracy to distinguish between false positive and true positive. Furthermore, MD simulations improved the accuracy of the results.

Moreover, a set of specific criteria have been proposed for compounds with potential antimalarial activity. These criteria postulate, on the beginning of development, an acceptable in vitro response and conformity to Lipinski’s ‘rule of five’. These are all present for Tx001, indicating that it could be the new lead compound. Finally, the data suggest that Tx001 probably acts by inhibiting PfATP6.

The continuous progress of the computational chemistry employed here is important for further antimalarial drug development. Further ligand optimisation cycles are needed to generate new prospects for docking and biological assays.
